# A permutation-based non-parametric analysis of CRISPR screen data

**DOI:** 10.1186/s12864-017-3938-5

**Published:** 2017-07-19

**Authors:** Gaoxiang Jia, Xinlei Wang, Guanghua Xiao

**Affiliations:** 1 Department of Statistical Science, Southern Methodist University, Dallas, TX 75205 USA; 2 Quantitative Biomedical Research Center, Department of Clinical Sciences, University of Texas Southwestern Medical Center, Dallas, TX 75390 USA; 3 Department of Bioinformatics, University of Texas Southwestern Medical Center, Dallas, TX 75390 USA; 4 Simmons Comprehensive Cancer Center, University of Texas Southwestern Medical Center, Dallas, TX 75390 USA

**Keywords:** Functional genomics, False discovery rate, RNA interference, Negative selection, Next generation sequencing, Positive selection

## Abstract

**Background:**

Clustered regularly-interspaced short palindromic repeats (CRISPR) screens are usually implemented in cultured cells to identify genes with critical functions. Although several methods have been developed or adapted to analyze CRISPR screening data, no single specific algorithm has gained popularity. Thus, rigorous procedures are needed to overcome the shortcomings of existing algorithms.

**Methods:**

We developed a Permutation-Based Non-Parametric Analysis (PBNPA) algorithm, which computes *p*-values at the gene level by permuting sgRNA labels, and thus it avoids restrictive distributional assumptions. Although PBNPA is designed to analyze CRISPR data, it can also be applied to analyze genetic screens implemented with siRNAs or shRNAs and drug screens.

**Results:**

We compared the performance of PBNPA with competing methods on simulated data as well as on real data. PBNPA outperformed recent methods designed for CRISPR screen analysis, as well as methods used for analyzing other functional genomics screens, in terms of Receiver Operating Characteristics (ROC) curves and False Discovery Rate (FDR) control for simulated data under various settings. Remarkably, the PBNPA algorithm showed better consistency and FDR control on published real data as well.

**Conclusions:**

PBNPA yields more consistent and reliable results than its competitors, especially when the data quality is low.

R package of PBNPA is available at: https://cran.r-project.org/web/packages/PBNPA/.

**Electronic supplementary material:**

The online version of this article (doi:10.1186/s12864-017-3938-5) contains supplementary material, which is available to authorized users.

## Background

The CRISPR (clustered regularly-interspaced short palindromic repeats) interference technique is widely used in biomedical studies to investigate gene functions. Large-scale screening with this technique has become a powerful tool in identifying cancer-promoting genes, drug-resistant genes, and genes that play pivotal roles in various biological processes [1–3]. The CRISPR/Cas9 system is composed of sgRNAs (single guide RNA) and Cas9s (CRISPR associated protein 9); an sgRNA contains around a 20-bp guide sequence that complements a DNA sequence and thus targets a gene of interest, and a Cas9 is a nuclease that induces double-strand breaks in the DNA and results in non-homologous end joining (NHEJ) repair. NHEJ is an error-prone repair mechanism that usually introduces an indel mutation that is highly likely to cause a coding frameshift, which leads to a premature stop codon and initiates the nonsense-mediated decay of the transcribed mRNA [1]. Thus, the CRISPR system abolishes the gene function by interfering with gene expression from the DNA level. This is more powerful than siRNA (small interfering RNA) or shRNA (short hairpin RNA) screens. An siRNA contains 20 ~ 25 bp short synthesized RNAs that function in the RNA interference pathway, and it cannot be integrated into a host genome. An shRNA contains synthesized double-stranded RNA molecules with a tight hairpin turn, which its plasmid vecto﻿r can be integrated into a host genome; however, it inhibits the gene function at the mRNA level [4]. All three types of screens are usually implemented on cultured cells: siRNA screens are carried out in multi-well plates with each well containing one or several siRNAs targeting the same gene, and the signal in each well is collected as the read for that well; by contrast, CRISPR and shRNA screens are carried out in a pooled manner, where a mixture of lentivirus that contains RNAi reagents (plasmid vector for either shRNA or sgRNA) targeting different genes is transfected into the same plate of cultured cells, and the microarray or next generation sequencing (NGS) technique can be used to collect reads. Cas9-sgRNA screens are performed with pre-designed sgRNA libraries that contain sgRNA redundancy. Generally, multiple sgRNAs (usually ranging from 3 to 10) with different sequences that target distinct locations on the same gene are utilized to ensure screening accuracy [1]. All genome-wide CRISPR screens use cell growth as a phenotypic measure. Based on the goal of the screens, they can be divided into positive selection screens and negative selection screens [5]. Positive screens aim to identify genes that inhibit cell growth in certain circumstances or that sensitize cells to a drug treatment or toxin. For example, genes upon ablation protecting cells against toxins, which are likely to be receptors for the toxins, or genes involved in downstream signaling pathways [6], may be targeted by positive screens. Under a strong selective pressure, cells with sgRNAs that confer resistance against that pressure would be enriched, and thus their signals are often strong and easy to detect. Negative selection screens aim to identify genes that promote cell growth or housekeeping genes [7]. In this scenario, cells that carry sgRNAs targeting such genes will be depleted during selection. Signals from negative screens are typically not as strong as those from positive screens, because the depletion level is usually mild and the number of depleted sgRNAs is large when considering the number of housekeeping genes (and thus they can be hard to separate from the background).

There are existing methods that can be used to analyze genome-wide RNA interfering screening results, including RSA [8], RIGER [9], MAGeCK [10], ScreenBEAM [11], etc. The Redundant siRNA Activity (RSA) method was originally developed to analyze data generated by large-scale small interfering RNA (siRNA) screens in mammalian cells [8]. RSA calculates a *p*-value for each gene based on an iterative hypergeometric distribution formula, where a smaller *p*-value indicates the gene is more likely to have higher activity. RNAi Gene Enrichment Ranking (RIGER) was originally designed to identify essential genes in genome-scale pooled shRNA screens [9]. It calculates the rank of each sgRNA based on a signal-to-noise metric and then synthesizes information on sgRNAs targeting the same gene in a way similar to that of Gene Set Enrichment Analysis to rank genes [12]. Model-based Analysis of Genome-wide CRISPR/Cas9 Knockout (MAGeCK) and Screening Bayesian Evaluation and Analysis Method (ScreenBEAM) were both designed to analyze CRISPR screen data. MAGeCK evaluates sgRNAs based on *p*-values calculated from fitting a negative binomial model [10], and then the ranks of sgRNAs targeting the same gene are combined with a modified version of robust ranking aggregation (RRA) called *α*-RRA. ScreenBEAM assesses the gene level activity with Bayesian hierarchical models [11], in which within-gene variances were modeled as random effects. Among the above methods, RIGER, MAGeCK and ScreenBEAM can perform both positive and negative selection. In addition, several algorithms used for analysis of Next Generation Sequencing (NGS) data, such as edgeR [13], DESeq [14] baySeq [15], NOISeq [16] and SAMseq [17], can also be used to analyze RNAi screening data. Although such methods can only assign ranks at the sgRNA level, they can be used to conduct gene-level inference [10] when combined with existing methods of integrating group information. It is worth noting that NOISeq and SAMseq both take nonparametric approaches. Unlike our method that is based on permutation, SAMseq mainly relies on the two-sample Wilcoxon statistic to estimate the significance; and NOISeq assesses the significance of the treatment effect with the reference distribution generated by comparing reads of each gene in samples under the same condition.

Although many CRISPR screen analysis methods are available, no single specific algorithm has gained popularity from researchers, mainly due to one or more of the drawbacks listed below: (1) Distributions assumed are doubtful or incorrect and thus incapable of modeling data variability from different sources. Researchers generally use negative binomial or Poisson distributions to model read counts from NGS [18]. However, these distributions do not reflect certain characteristics of NGS data generating processes and are weak in handling over-dispersion. (2) Most studies compared their model performance using some ‘oracle’ datasets. However, the performance may be compromised when generalizing these methods to datasets from different conditions or platforms. This is reflected by the fact that the number of consistently identified genes across different algorithms is often small [19]. (3) Published methods usually have loose or no false discovery rate (FDR) control. FDR reflects the rate of type I errors when performing multiple hypothesis tests and influences the credibility of the tests if not carefully controlled. False discovery is a big concern for functional genomic studies when a large number of statistical tests are performed [20]. The above-mentioned methods tend to overlook FDR or be ineffective in controlling it, as will be shown in detail in the Results section. Without stringent FDR controlled *p*-values, it is difficult to evaluate the statistical significance of selected genes.

Our proposed method, Permutation-Based Non-Parametric Analysis (PBNPA) of CRISPR screen data, mitigates the three major drawbacks of existing CRISPR methods. First, PBNPA computes *p*-values at the gene level by permuting sgRNA labels, and thus it avoids restrictive distributional assumptions. Second, PBNPA shows superior performance to other algorithms in simulation using data generated to mimic the NGS sequencing process, which avoids overfitting based on specific datasets. Application to real data confirms better consistency of PBNPA. Last, our data application reveals that PBNPA outperformed its competitors in terms of FDR control.

## Methods

### A permutation procedure

In a CRISPR screen dataset, assume *Y*_*ij*_ is the read count for the *j*th sgRNA in the library under condition *i*, where *j* = 1 , 2 ,  …  , *J* indexes sgRNAs in the library; and *i* = 0 , 1 indexes two experimental conditions, with *i* = 0 for the control and *i* = 1 for the treatment. We use *I*_*g*_ to denote the index set of the sgRNAs that target the same gene *g* and ${\bigcup}_{g=1}^G{I}_g=\left\{1,2,\dots, J\right\}$, where *g* = 1 , 2 ,  …  , *G* and *G* is the total number of genes in the library. Raw read counts in each condition *i* were normalized by multiplying a factor of $mean\ \left({\sum}_{j=1}^J{Y}_{0 j},{\sum}_{j=1}^J{Y}_{1 j}\right)/{\sum}_{j=1}^J{Y}_{ij}$. This makes total read counts in each condition equal without losing any useful information. Our PBNPA algorithm is outlined below.

1. For each sgRNA *j* (*j* = 1, 2, …*J*), calculate the natural logarithm fold change of normalized read counts: ${r}_j= \log\ \left(\frac{Y_{1 j}}{Y_{0 j}}\right)$. Then for each gene g, use the median of *r*_*j*_’s (*j* ∈ *I*_*g*_) as the *R* score, denoted by *R*_*g*_.
2. Randomly permute gene labels while holding (*Y*_0*j*_, *Y*_1*j*_) pairs unchanged to get permutated *R* scores for each gene, denoted by ${R}_{g1}^{\ast }$’s, where *g* = 1 , 2 ,  …  , *G*.
3. Repeat step 2 for *T* times and pool all ${R}_{gt}^{\ast }$’s over the *T* permutations and all genes to form a null distribution of *R*.
4. Calculate the *p* value for gene *g* if it is a positively selected gene as:

$$ p=\frac{\# of\  permuted\  R\  scores>{R}_g}{total\# of\  permuted\  R\  scores} $$;

and the *p* value for gene *g* if it is a negatively selected gene as:

$$ p=\frac{\# of\  permuted\  R\  scores<{R}_g}{total\# of\  permuted\  R\  scores} $$;.

5. After getting *p* values for all genes, remove genes with *p* values smaller than a threshold, which are considered to be significant genes. Then repeat step 2 and 3 to get the null distribution with significant genes removed. Get updated *p* values for each gene as described in step 4.
6. Use the Benjamini-Hochberg procedure to control FDR [21].

In this algorithm, the median log fold change of sgRNAs targeting a gene is used as the *R* score of that gene, which makes it more robust against any outliers and influences from potential off-target effects. In step 5, we remove a small portion of genes with the purpose of removing any significant genes to get a more accurate estimate of the null distribution [22], as the null distribution is likely to be distorted if these significant genes are kept in the permutation process.

### Simulation strategy

To mimic the nature of RNA-seq experiments, the read counts of all sgRNAs under a given condition were generated from a Dirichlet-multinomial (DM) distribution. Considering the experimental setup of CRISPR screening with RNA-seq, each sgRNA in a library can be viewed as an outcome category in a multinomial distribution when the total read count (sequencing depth) is fixed. However, the literature indicates that multinomial distributions are inadequate to model the extra variability that is usually observed in NGS data [23, 24]. To account for over-dispersion, the probability vector of an NGS read falling into the different sgRNA categories is modeled as random variables from a Dirichlet distribution. After combining the multinomial model with the Dirichlet model, the mixture model is a Dirichlet-multinomial model with the probability mass function (PMF) shown below:

$$ f\left({\boldsymbol{Y}}_{\boldsymbol{i}}\right)=\frac{\varGamma \left({Y}_{i+}+1\right)\varGamma \left({\gamma}_{i+}\right)}{\varGamma \left({Y}_{i+}+{\gamma}_{i+}\right)}\prod_{j=1}^J\frac{\varGamma \left({Y}_{i j}+{\gamma}_{i j}\right)}{\varGamma \left({Y}_{i j}+1\right)\varGamma \left({\gamma}_{i j}\right)} $$

where ***Y***_***i***_ = [*Y*_*i*1_, *Y*_*i*2_,  … , *Y*_*iJ*_], ${Y}_{i+}={\sum}_j^J{Y}_{i j}$, ${\gamma}_{i+}={\sum}_j^i{\gamma}_{i j}$ with *γ*_*ij*_’s being the parameters of the DM distribution; and $E\left({Y}_{i j}\right)={Y}_{i+}\frac{\gamma_{i j}}{\gamma_{i+}}$ and $Var\left({Y}_{i j}\right)={Y}_{i+}\frac{\gamma_{i j}}{\gamma_{i+}}\left(1-\frac{\gamma_{i j}}{\gamma_{i+}}\right)\left(\frac{Y_{i+}+{\gamma}_{i+}}{1+{\gamma}_{i+}}\right)$ [23, 25]. Compared to the variance of the multinomial model, the variance of the DM model is increased by a factor of $\left(\frac{Y_{i+}+{\gamma}_{i+}}{1+{\gamma}_{i+}}\right)$. When the total read count *Y*_*i*+_ is fixed, *γ*_*i*+_ controls the degree of overdispersion with a smaller value indicating larger overdispersion.

To simulate read counts for a screen experiment, we first generated *γ*_0*j*_’s for a control sample from a negative binomial distribution *NB* (*q*, *p*) where *q* is the number of successful trials to be reached and *p* is the probability of success in each trial. We set *q* = 3 and *p* = 0.08 so that the generated DM read counts are right skewed, which approximately mimics real data. We link *γ*_*ij*_ to the effect of sgRNA *j* through the relationship *γ*_*ij*_ = exp (*α*_*j*_ + *β*_*j*_ × *i*), where *α*_*j*_ loosely reflects the log mean read count under the control and *β*_*j*_ represents the *j*th sgRNA effect (i.e., the log difference in mean read count between the treatment and control). The total number of genes *G* was set to be 10,000. For genes that have effects during the screen processes under different conditions (which are referred to as true hits), we first generated the sgRNA effects targeting gene *g* from a normal distribution, *β*_*j*_ ~ *N* (*μ*_*g*_, *σ* ^2^) for *j* ∈ *I*_*g*_ , *g* = 1 , 2 ,  …  , *G*, with gene-specific mean *μ*_*g*_ and constant standard deviation *σ* = 0.4 (0.4 was chosen to be close to the standard deviation estimated from real data); and then we forced all *β*_*j*_’s for gene *g* to have the same sign as *μ*_*g*_. The vector, which contains different levels of *μ*_*g*_ in our simulation, was set to be [1.5, 1, 0.5, −1, −2, −3], where a positive number indicates that a gene’s ablation promotes cell growth while a negative number indicates a gene is necessary for cell growth. The three levels of *μ*_*g*_ for each sign represent the high/medium/low effects of positively/negatively selected genes, respectively. There are 50 genes simulated from each level of *μ*_*g*_. Thus, among the 10,000 genes, there are 150 positively selected genes and 150 negatively selected genes. For those genes with no effects, *β*_*j*_’s were set to be 0.

Off-target effects of CRISPR are often caused by unintended DNA cleavage at non-targeting sites as a result of mismatch between DNA and sgRNA [26]. If an sgRNA is an off-target effect, its read count may either decrease, increase, or remain the same since most DNA sequences in the human genome have no known function. In our simulation, off-target *β*_*j*_’s were simulated from *N*(0, *σ* ^2^) and then used to replace a certain proportion of randomly-selected on-target sgRNAs. The off-target rate of a library can be considered an important characteristic reflecting the quality of the library, which is determined by the algorithm used to design the sgRNAs [27]. Although several experimental approaches exist, it is still challenging to get accurate estimates of sgRNA off-target rates [28, 29]. Reported off-target rates vary greatly in the literature [30, 31] and can range between 1% and 20% in most sgRNA libraries. Thus, we tested 4 off-target proportion values: 1%, 5%, 10% and 20%, to represent sgRNA libraries of different quality.

Besides the library quality, the number of sgRNAs per gene is another factor that is known to influence the screen performance dramatically. Thus, we varied the number of sgRNAs per gene from 2 to 6 as well.

With *β*_*j*_’s simulated for all sgRNAs, we obtained *γ*_1*j*_ = *γ*_0*j*_ exp (*β*_*j*_). Then we simulated *Y*_*ij*_ from the DM distribution with *γ*_*ij*_’s from statistical packages ‘multinomRob’ [32] and ‘dirmult’ [33].

### Combining *p*-values to handle replicates

A CRISPR screen experiment may contain several replicates. We analyzed each replicate using the proposed algorithm and then employed Fisher’s method to combine *p*-values from replicates for each gene [34, 35]. According to Fisher’s method, the statistic $-2{\sum}_{s=1}^S \ln {p}_{gs}$, with *p*_*gs*_ representing gene *g*’s *p* value from the *s*th replicate, follows an *χ* ^2^ distribution with 2*S* degrees of freedom under the null hypothesis *H*_0_: gene *g* has no effect, from which a combined *p* value for each gene *g* is obtained [34].

## Results

### Positive selection performance

**Fig. 1:**
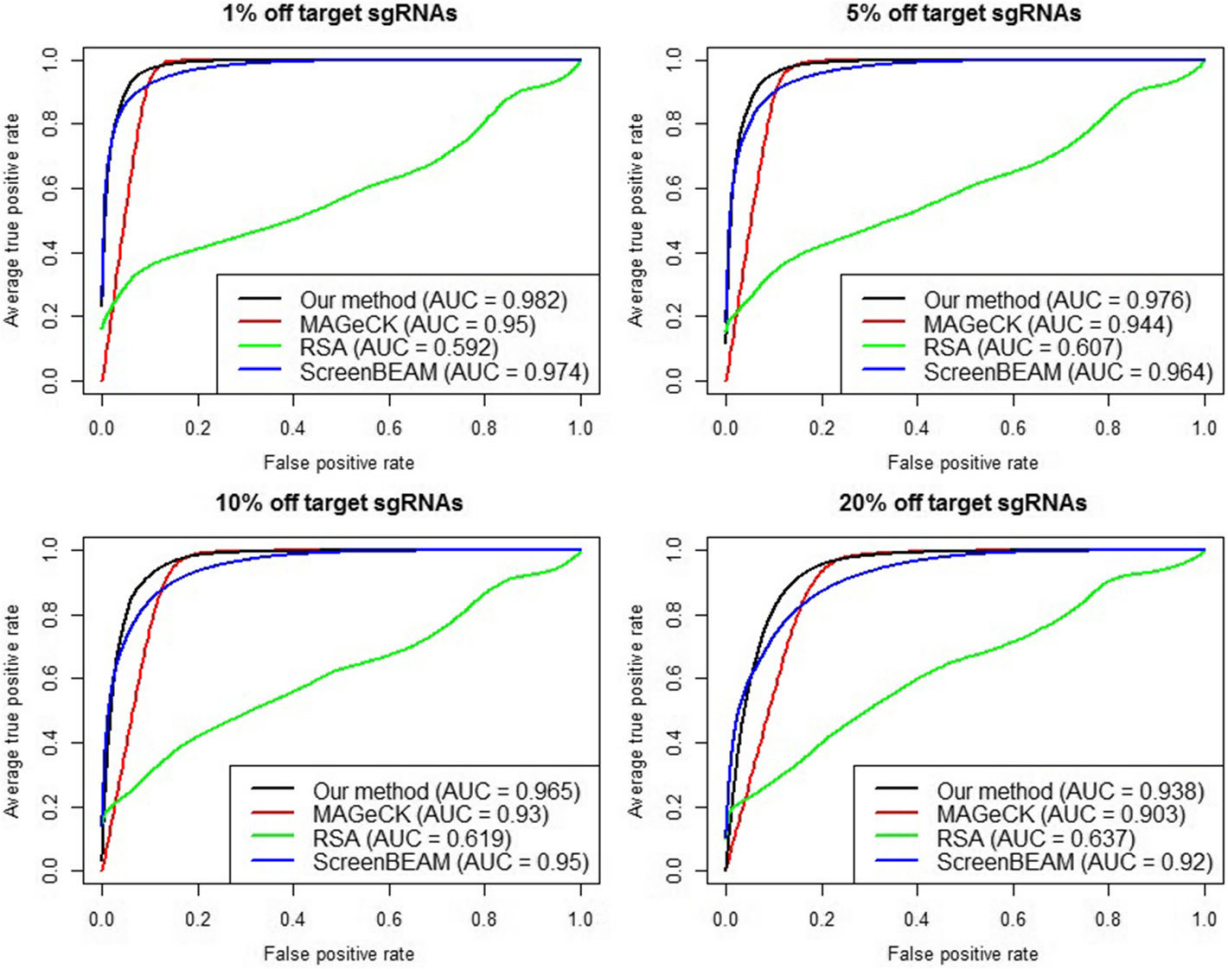
Simulation evaluation of positive selection performance. ROC curves and AUCs are shown for different algorithms with an increasing off target proportion while the number of sgRNAs per gene is fixed at 3. Each curve represents the average of ROC curves for 50 simulated datasets and above

**Fig. 2:**
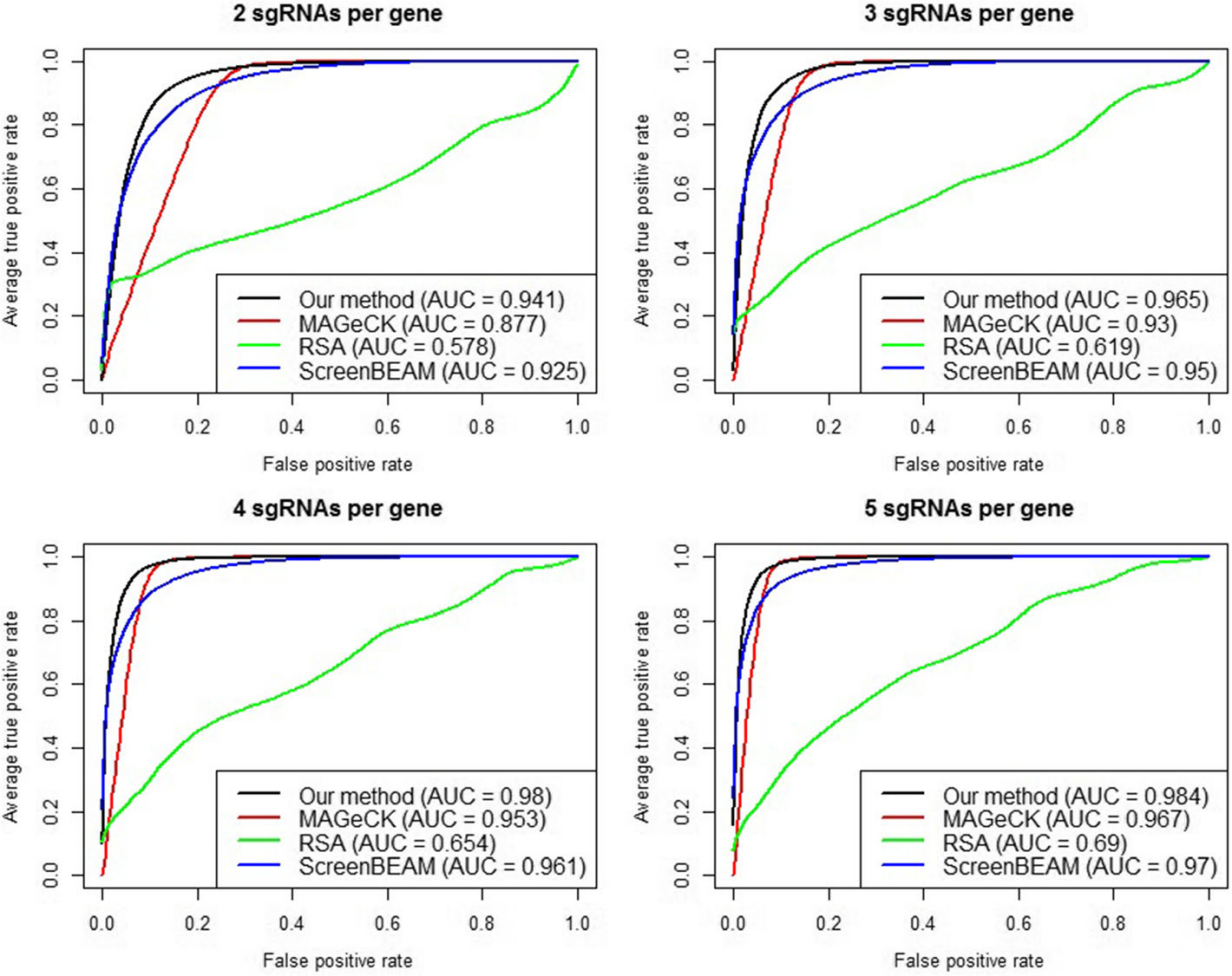
Simulation evaluation of positive selection performance. ROC curves and AUCs are shown for different algorithms with an increasing number of sgRNAs per gene, while the off target proportion is fixed at 10%

We compared the performance of PBNPA, RSA, ScreenBEAM and MAGeCK for the four different off-target rates (1%, 5%, 10%, 20%), as mentioned in the simulation strategy section, when there are 3 sgRNAs targeting each gene. A receiver operating characteristic (ROC) curve plots the true positive rate against the false positive rate of a binary classifier for different possible cut-off points and visualizes the performance of the classifier. As shown in Fig. 1, PBNPA works better for positive screening than RSA, MAGeCK and ScreenBEAM in terms of the ROC curve and area under the curve (AUC), regardless of the off-target proportion. Also, all the algorithms show worse performance with an increasing off-target rate except for RSA, whose AUC increases from 0.592 to 0.637. Figure 2 indicates that PBNPA outperforms the other algorithms with varying numbers of sgRNAs per gene from 2 to 5. As expected, the AUC of each method increases with an increasing number of sgRNAs per gene, as more sgRNAs enable better estimation of gene effects.

As we have discussed previously, *γ*_*i*+_ controls the degree of overdispersion. To check the performance of the algorithms with an increased overdispersion level, we divided every *γ*_*ij*_ by 10 and report the results in Figures S1 and S2 of Additional file 1: the performance of nearly all algorithms decreases compared with the low overdispersion setting, but the performance of PBNPA and ScreenBEAM is comparable, and it is better than RSA and MAGeCK.

### Negative selection performance

**Fig. 3:**
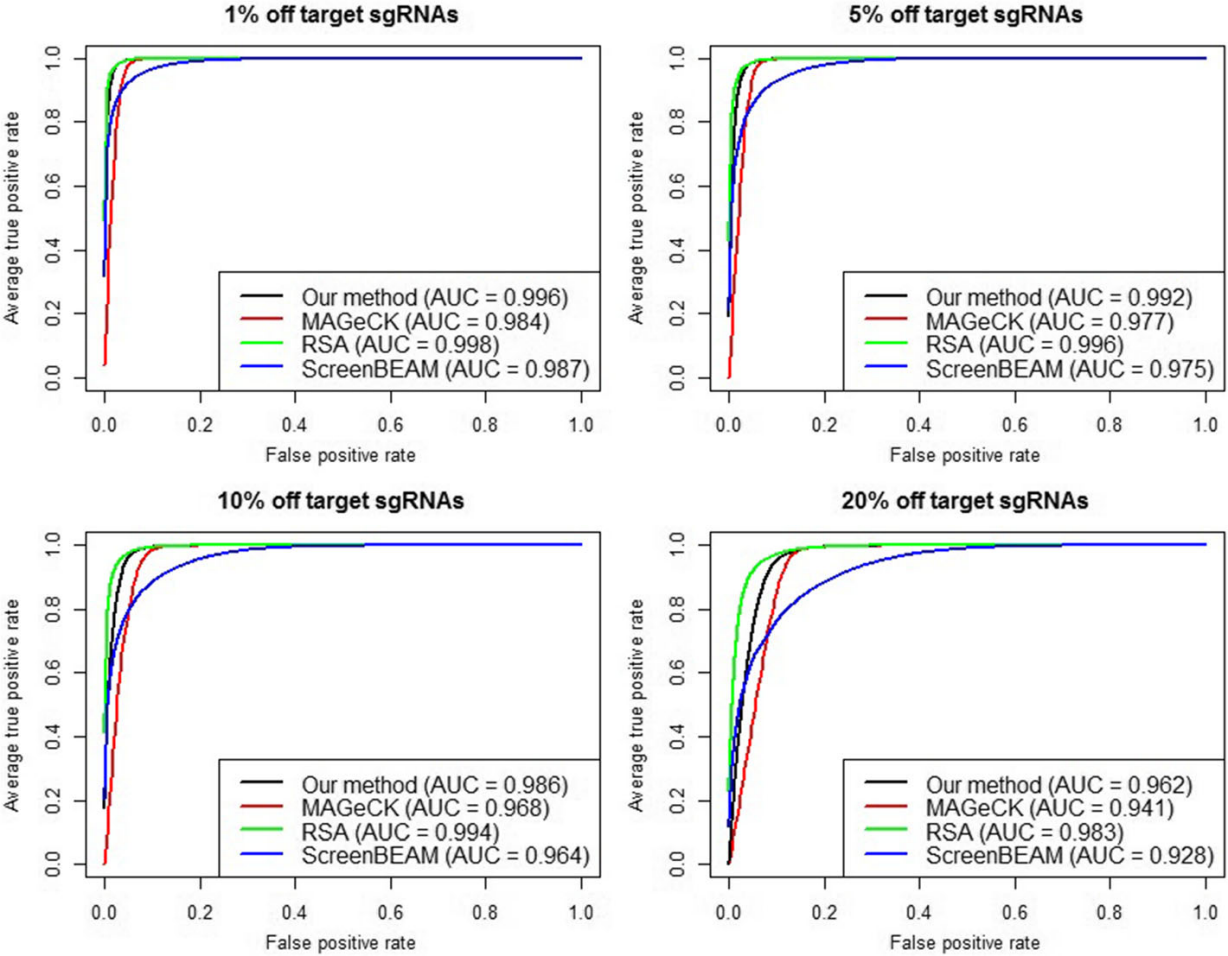
Simulation evaluation of negative selection performance. ROC curves and AUCs are shown for different algorithms with an increasing off target proportion, while the number of sgRNAs per gene is fixed at 3

**Fig. 4:**
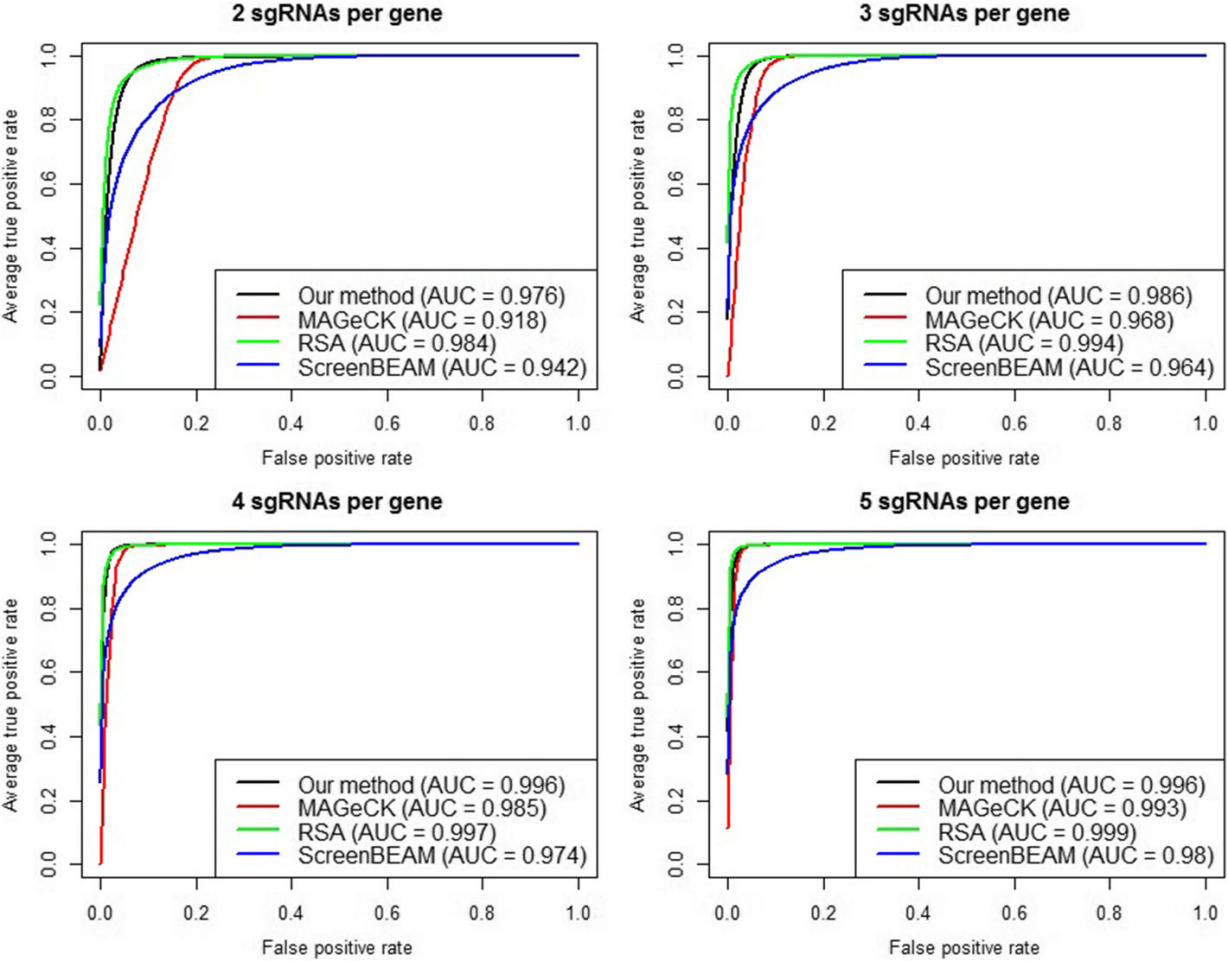
Simulation evaluation of negative selection performance. ROC curves and AUCs are shown for different algorithms with an increasing number of sgRNAs per gene, while the off target proportion is fixed at 10%

For negative selection, PBNPA and RSA have similar AUCs and perform better than MAGeCK and ScreenBEAM when the proportion of off-target sgRNAs is low, as shown in Fig. 3. When the proportion of off-target sgRNAs increases, RSA shows some advantage over PBNPA and is robust against this increase. Figure 4 shows that when we fix the off-target proportion at 10% and vary the number of sgRNAs per gene, PBNPA and RSA have comparable performance, and they are significantly better than MAGeCK and ScreenBEAM when the number of sgRNAs per gene is low.

In the setting of high overdispersion, RSA is the best among all and PBNPA is only second to RSA with increasing off-target proportion in the simulated datasets, as shown in Figure S3 (Additional file 1). Figure S4 (Additional file 1) shows that when we fix the off-target proportion and vary the number of sgRNAs per gene, RSA is slightly better than PBNPA, and they are better than the other two algorithms across different numbers of sgRNAs per gene. Overall, for negative selection, RSA seems to be the winner; but PBNPA provides quite close or comparable performance to RSA, which is much better than MAGeCK and ScreenBEAM.

### Comparison of recall, precision and estimation of *p* values

**Fig. 5:**
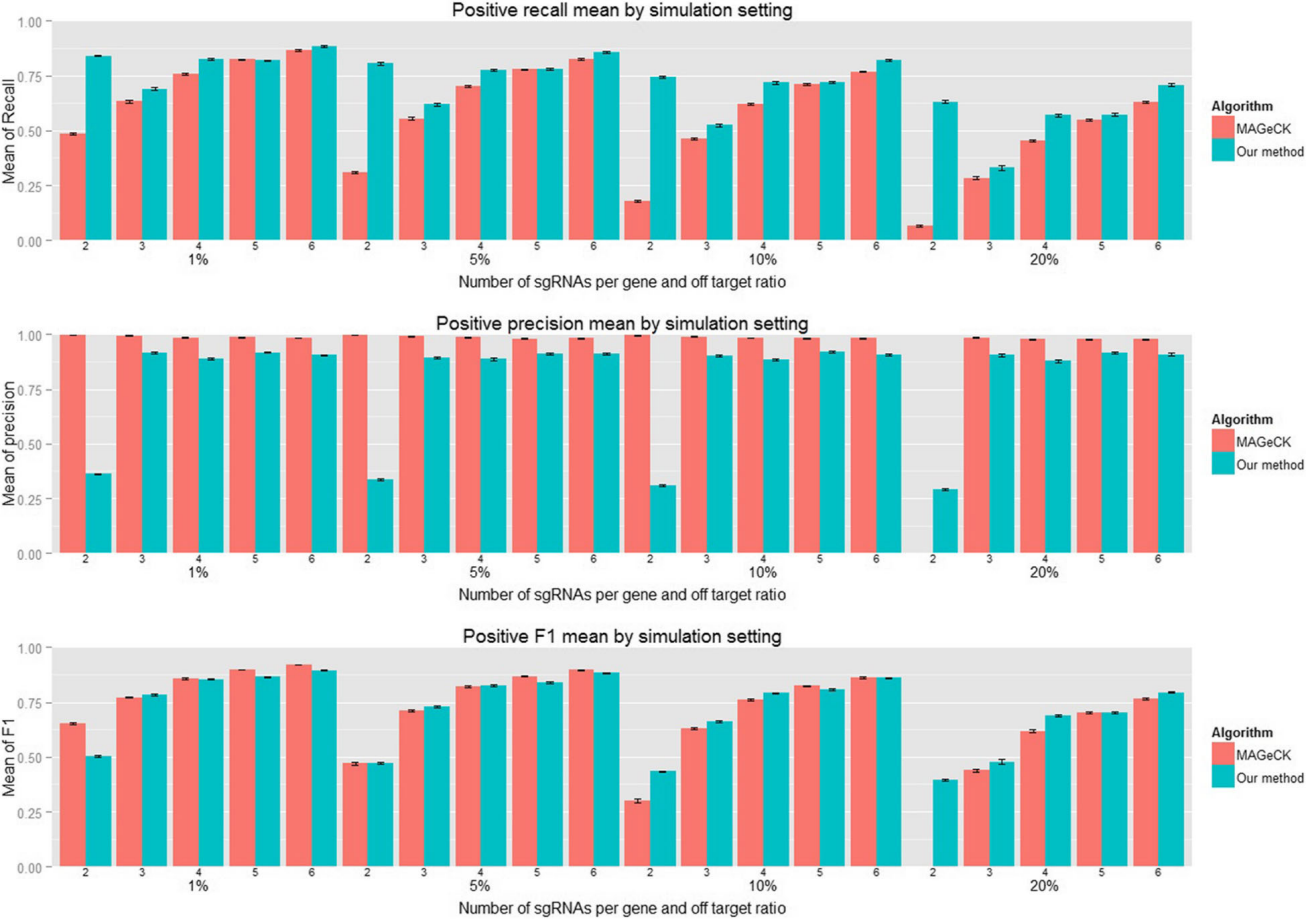
Simulation evaluation of positive selection performance based on recall, precision and *F*_1_ for different combinations of sgRNA number per gene (2 ~ 6) and off target ratio. Each bar represents the average of 50 simulated datasets and the standard error is indicated on the bar

When multiple statistical tests are performed simultaneously in the analysis of a dataset, adjustment of *p* values is needed. Among the four algorithms, RSA does not provide a method to adjust for multiple comparison. We applied the Benjamini-Hochberg (BH) procedure [21] to the results from RSA and obtained FDR-adjusted *p* values. The other three methods use the BH procedure by default. Then we controlled FDR at 5% and compared recall (percent of identified true hits among all true hits), precision (percent of identified true hits among all selected genes) and *F*_1_ of the four algorithms, where *F*_1_ is a metric that balances recall and precision and is defined as ${F}_1=2\times \frac{recall\times precision}{recall+ precision}$. To our surprise, when FDR was controlled at 5%, neither RSA nor ScreenBEAM was able to identify any significant genes. Actually, under most settings, all genes in the RSA results had an adjusted *p*-value of 1. This suggests that RSA and ScreenBEAM cannot accurately estimate the statistical significance of the genes. Thus, we compared the recall, precision and *F*_1_ of PBNPA and MAGeCK. Figure 5 shows the recall, precision and *F*_1_ of PBNPA and MAGeCK for different combinations of sgRNA number per gene (2, 3, 4, 5, 6) and off-target rates (1%, 5%, 10%, 20%) for positive screens. From the bottom panel of Fig. 5, it is clear that under most settings, *F*_1_ of PBNPA is the same as or slightly better than that of MAGeCK. However, the recall of PBNPA is significantly better than that of MAGeCK, especially when the number of sgRNAs per gene is small. In the middle panel, MAGeCK consistently maintains very high precision across all the settings. However, MAGeCK tends to be too conservative in identifying true hits and may show a lack of power. Note that when the off-target rate is high (20%) with 2 sgRNAs per gene, MAGeCK has a recall rate of less than 10%, where it cannot identify any true hits at all in some simulated datasets. In screening experiments, after the genome-wide screening, a secondary screening will typically be used to validate hits from the first round [36]. This highlights the importance of the recall rate: those false positives are likely to be removed in the secondary screening, while those false negatives can be crucial genes that will be missed permanently. Nearly the same pattern can be observed for negative screens, as shown in Figure S5 (Additional file 1). Thus, PBNPA provides the most accurate estimation of adjusted *p* values among the four algorithms and also offers optimal recall rates.

### Handling replicates

The comparisons we have discussed above are based on simulated data with no replicates. For low-quality screens, replicates are typically used to increase the power of identification. To handle screens with replicates, we propose to use Fisher’s method to combine *p* values, as mentioned in the Methods section, followed by FDR adjustment. We simulated replicate datasets with parameters of the DM distribution set as $\frac{\gamma_{ij}}{5}$, which has higher overdispersion than the DM distribution with *γ*_*ij*_ and so may represent data of low quality. We evaluated 3 simulated replicates independently. Among the 150 positively selected genes, the analysis of individual replicates gives the following results (i.e., number of true hits identified/number of genes identified by PBNPA) with FDR controlled at 5%: 6/7, 9/11, and 8/9, respectively. After combining *p* values for the first two replicates, the result is 72/86. After combining *p* values for all three replicates, the result is 96/111. It is evident that PBNPA shows dramatically improved performance when even a small number of replicates are present.

### Comparison using real data

Although the performance of various algorithms usually does not differ greatly in simulation studies, they tend to give quite different inferences on real data. This can be due to the fact that a simulation is not an exact reproduction of the complex data generation process in the real world. This phenomenon is also observed in algorithms analyzing CRISPR data [19]. From the simulation study, we have found that PBNPA and MAGeCK are handy to use and give better overall performance than the other two algorithms. Thus, we used datasets from two recently published articles to evaluate the consistency between these two algorithms as well as the consistency of the same algorithm on different replicates from the same experiment, since a good algorithm should give highly similar results on replicates of the same experiment. Control of FDR is also studied by comparing control vs control or treatment vs treatment read counts between replicates, as no genes should be identified in this comparison.

**Fig. 6:**
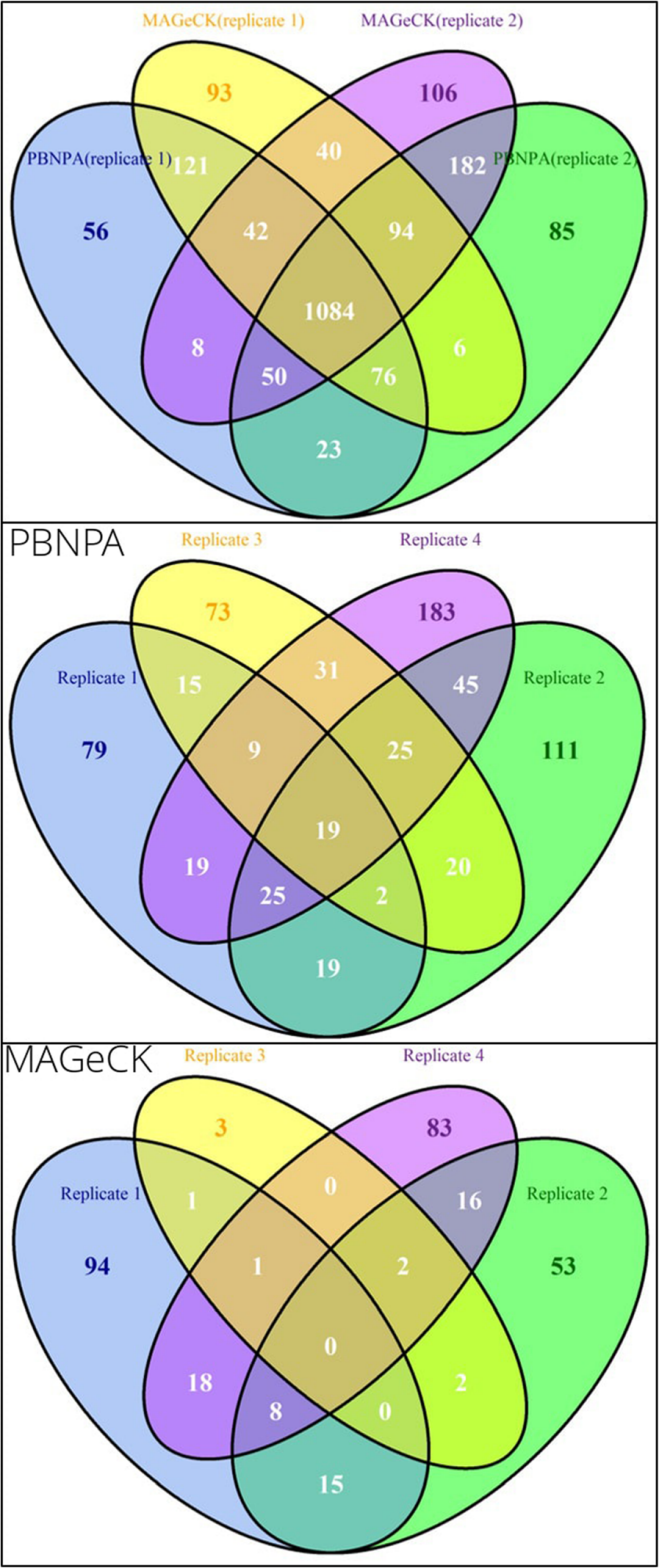
Comparing consistency of MAGeCK and PBNPA on replicates using real data. *Upper panel*: overlap of PBNPA and MAGeCK results on replicates 1 and 2 of the KBM7 dataset. *Middle panel*: overlap of PBNPA results on the four replicates of Toxoplasma. *Bottom panel*: overlap of MAGeCK results on the four replicates of Toxoplasma

The KBM7 dataset is from a study with two replicates and 10 sgRNAs per gene, which aims to identify essential genes in the human genome to reveal genes that are oncogenic drivers or lineage specifiers [37]. We analyzed controls vs controls or treatment vs treatment with the two algorithms and found that PBNPA has fewer falsely identified genes compared with MAGeCK, as shown in Table 1. As shown in the upper panel of Fig. 6, the identified hits are highly overlapped between the two algorithms for the same replicate, as well as between the two replicates with the same algorithm. This indicates both algorithms perform well on this dataset with high consistency.

**Table 1:** Comparison of FDR control between MAGeCK and PBNPA

Dataset		KBM7		Toxoplasma			
Selection direction	Algorithm	Ctrl1 vs ctrl2	Treat1 vs treat2	Ctrl1 vs ctrl2	Ctrl1 vs ctrl3	Treat1 vs treat2	Treat1 vs treat3
Positive	MAGeCK	50	18	0	1	0	1
	PBNPA	38	10	0	1	1	0
Negative	MAGeCK	0	3	4	2	6	28
	PBNPA	0	6	0	2	0	0

The Toxoplasma dataset is from a study with four replicates, which aims to identify essential genes of parasites for infection of human fibroblasts [38]. The library was designed to target more than 8000 protein coding genes in *T. gondii* with 10 sgRNAs per gene. The analysis with the two algorithms shows that PBNPA has fewer falsely identified genes than MAGeCK, as shown in Table 1. Furthermore, the number of consistently identified genes for PBNPA is significantly higher than that identified by MAGeCK among the 4 replicates, as is shown in the middle and bottom panels of Fig. 6. For PBNPA, there are 19 genes consistently identified in all four replicates and 80 genes consistently identified in at least three replicates. However, for MAGeCK there is no gene identified in all four replicates and only 11 genes consistently identified in at least three replicates. This is strong evidence that PBNPA has superior consistency and better FDR control than MAGeCK.

The similarities and differences in performance of the two algorithms on these two datasets can be explained below. In the KBM7 dataset, each gene is targeted by 10 sgRNAs. From our simulation study, 10 sgRNAs per gene should be sufficient to give reliable inference on the hits. Thus, these two algorithms give highly similar results. For the Toxoplasma dataset, although there are 10 sgRNAs designed for each gene, the algorithm used to design sgRNAs is optimized for human genes not for Toxoplasma, which, we conjecture, would deteriorate the efficiency of sgRNAs in the screen. In addition, the screening pipeline for Toxoplasma differs from that for cultured human cells, which may induce unknown variability in the data. Based on the above rationale, we conclude that PBNPA is more robust to data variability than MAGeCK.

Finally, we note that the other two methods did not perform well on these real data, which agrees with our findings from simulation. In particular, RSA showed poor performance in controlling FDR; for example, in the KBM7 dataset, when we compared ctrl1 vs. ctrl2, RSA claimed more than 90% of the genes are significant when controlling FDR at 5% for positive selection. This is also consistent with an observation in the MAGeCK paper [10] that RSA has a high FDR.

## Discussion

While researchers typically use gene-specific null distributions in their permutation procedures, we employed a common null probability distribution for all genes in PBNPA. We find that this gives similar or even slightly better performance than using gene-specific null distributions. However, building a common null distribution for all genes substantially saves computation time over building gene specific null distributions. For example: if there are 10,000 genes and we permute 10 times, we can get a common null distribution for all genes based on 10,000 × 10 = 100,000 replicates; but we need to permute 100,000 times if we want an individual null distribution for each gene based on the same number of replicates. Here, using a common null distribution saves 10,000 times as much computation time as using gene-specific null distributions.

Although our algorithm is designed to analyze CRISPR data, it can also be applied to analyze genetic screens implemented with siRNAs or shRNAs and drug screens, which all generate data with structures similar to those in CRISPR screens. The idea of doing permutation twice, with significant genes from the first round removed to get a more accurate null distribution, could be used by other studies where *p* values are mainly generated from a permutation process. We note that there are supervised methods of analyzing CRISPR data, which need previous knowledge to estimate the background noise in the platform and variability in the data [39]. Such methods are suitable in situations when reliable previous screening results are available.

## Conclusions

To the best of our knowledge, our paper is the first study to compare the performance of several algorithms with simulated datasets. With the known ground truth, we showed the overall superiority of our PBNPA algorithm compared to several existing methods in analyzing CRISPR data, which is also verified by the real data studies. The behaviors of each algorithm are revealed from simulation studies, which could help researchers select the most appropriate algorithm to analyze CRISPR data.

Although there are many existing algorithms available for analyzing CRISPR data, researchers are particularly interested in new algorithms that can give consistent and reliable results with a small number of sgRNAs per gene and a low sequencing depth and that are not sensitive to platforms, which will facilitate genome-scale screens while lowering the cost. Our PBNPA algorithm is a step toward achieving this goal.

## Additional file

Additional file 1: Figure S1. Simulation evaluation of positive selection performance using datasets with an increased overdispersion level. ROC curves and AUCs are shown for different algorithms with an increasing off target proportion while fixing the number of sgRNAs per gene at 3. Each curve represents the average of ROC curves for 50 simulated datasets and hereafter. **Figure S2.** Simulation evaluation of positive selection performance using datasets with an increased overdispersion level. ROC curves and AUCs are shown for different algorithms with an increasing number of sgRNAs per gene while fixing the off target proportion at 10%. **Figure S3.** Simulation evaluation of negative selection performance using datasets with an increased overdispersion level. ROC curves and AUCs are shown for different algorithms with an increasing off target proportion while fixing the number of sgRNAs per gene at 3. **Figure S4.** Simulation evaluation of negative selection performance using datasets with an increased overdispersion level. ROC curves and AUCs are shown for different algorithms with an increasing number of sgRNAs per gene while fixing the off target proportion at 10%. **Figure S5.** Simulation evaluation of negative selection performance based on recall, precision and *F*_1_ for different combinations of sgRNA number per gene (2 ~ 6) and off target ratio. Each bar represents the average of 50 simulated datasets and standard error is indicated on the bar. (DOCX 925 kb)

## Abbreviations

BH: Benjamini-Hochberg
CRISPR: Clustered regularly-interspaced short palindromic repeats
DM: Dirichlet-multinomial
FDR: False discovery rate
MAGeCK: Model-based Analysis of Genome-wide CRISPR/Cas9 Knockout
NGS: Next generation sequencing
NHEJ: Non-homologous end joining
PBNPA: Permutation-Based Non-Parametric Analysis
PMF: Probability mass function
RIGER: RNAi Gene Enrichment Ranking
ROC: Receiver operating characteristic
RRA: Robust ranking aggregation
RSA: Redundant siRNA Activity
ScreenBEAM: Screening Bayesian Evaluation and Analysis Method
sgRNA: Single guide RNA
shRNA: Short hairpin RNA
siRNA: Small interfering RNA
