# Supplementary material for: A permutation-based non-parametric analysis of CRISPR screen data
Source: BMC Genomics. 2017 Jul 19;18:545. doi: 10.1186/s12864-017-3938-5 (PMC5518132; doi:10.1186/s12864-017-3938-5)
Supplement: Additional file 1: Figure S1. — Simulation evaluation of positive selection performance using datasets with an increased overdispersion level. ROC curves and AUCs are shown for different algorithms with an increasing off target proportion while fixing the number of sgRNAs per gene at 3. Each curve represents the average of ROC curves for 50 simulated datasets and hereafter. Figure S2. Simulation evaluation of positive selection performance using datasets with an increased overdispersion level. ROC curves and AUCs are shown for different algorithms with an increasing number of sgRNAs per gene while fixing the off target proportion at 10%. Figure S3. Simulation evaluation of negative selection performance using datasets with an increased overdispersion level. ROC curves and AUCs are shown for different algorithms with an increasing off target proportion while fixing the number of sgRNAs per gene at 3. Figure S4. Simulation evaluation of negative selection performance using datasets with an increased overdispersion level. ROC curves and AUCs are shown for different algorithms with an increasing number of sgRNAs per gene while fixing the off target proportion at 10%. Figure S5. Simulation evaluation of negative selection performance based on recall, precision and F 1 for different combinations of sgRNA number per gene (2 ~ 6) and off target ratio. Each bar represents the average of 50 simulated datasets and standard error is indicated on the bar. (DOCX 925 kb) [file 12864_2017_3938_MOESM1_ESM.docx]

*Supplementary Material*
PBNPA: a permutation-based non-parametric analysis of CRISPR screen data

# Additional Simulation Results:

#
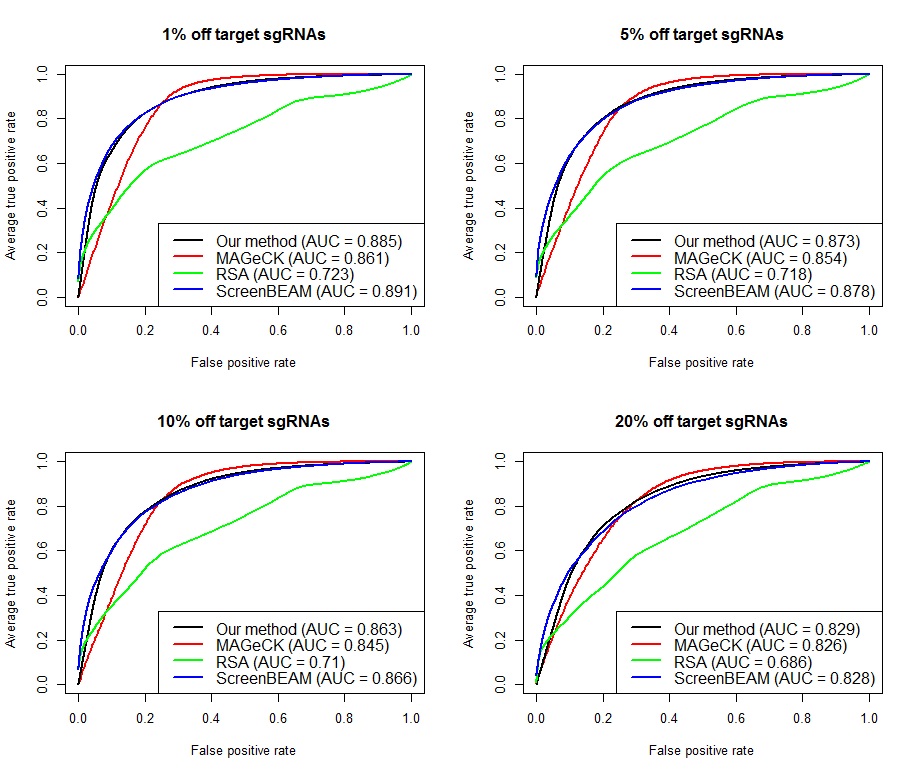


Figure S1 Simulation evaluation of positive selection performance using datasets with an increased overdispersion level. ROC curves and AUCs are shown for different algorithms with an increasing off target proportion while fixing the number of sgRNAs per gene at 3. Each curve represents the average of ROC curves for 50 simulated datasets and hereafter.


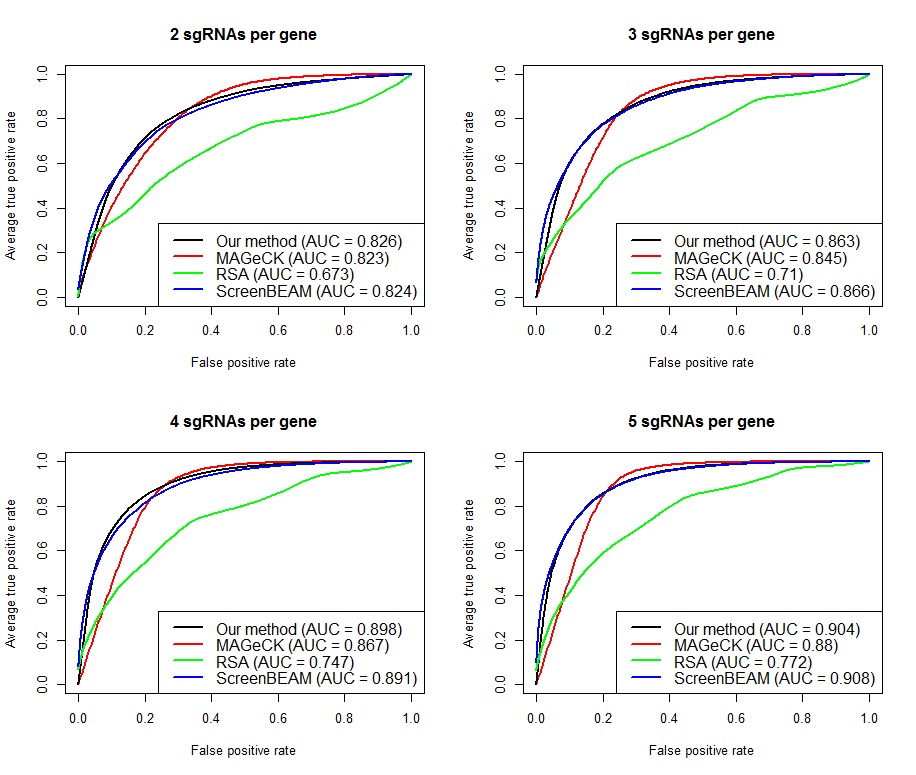


Figure S2 Simulation evaluation of positive selection performance using datasets with an increased overdispersion level. ROC curves and AUCs are shown for different algorithms with an increasing number of sgRNAs per gene while fixing the off target proportion at 10%.


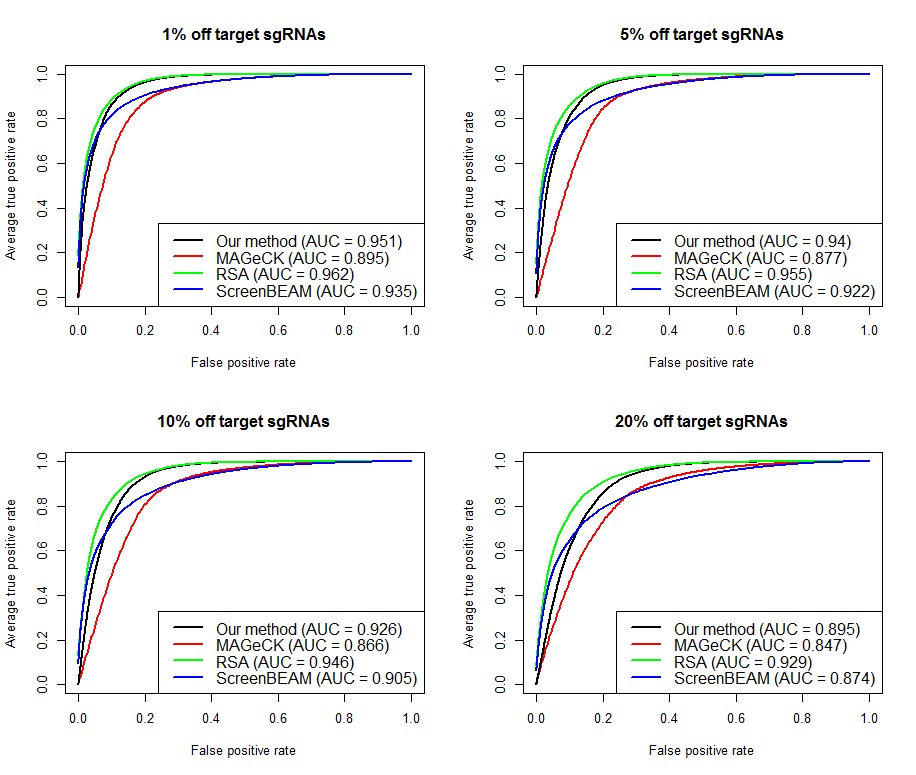


Figure S3 Simulation evaluation of negative selection performance using datasets with an increased overdispersion level. ROC curves and AUCs are shown for different algorithms with an increasing off target proportion while fixing the number of sgRNAs per gene at 3.


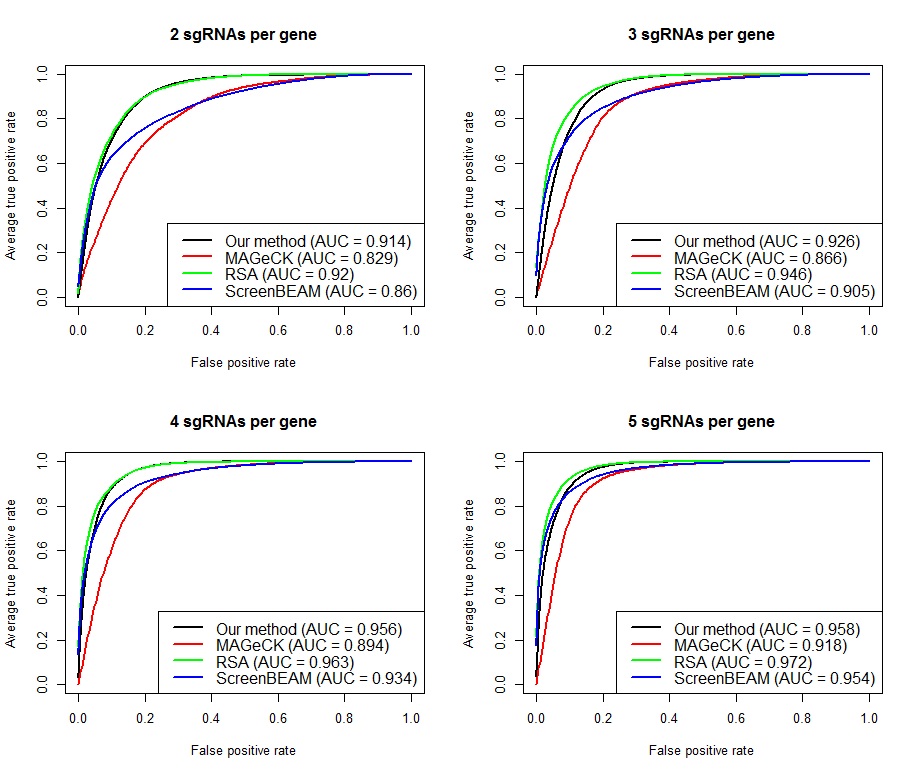


Figure S4 Simulation evaluation of negative selection performance using datasets with an increased overdispersion level. ROC curves and AUCs are shown for different algorithms with an increasing number of sgRNAs per gene while fixing the off target proportion at 10%.


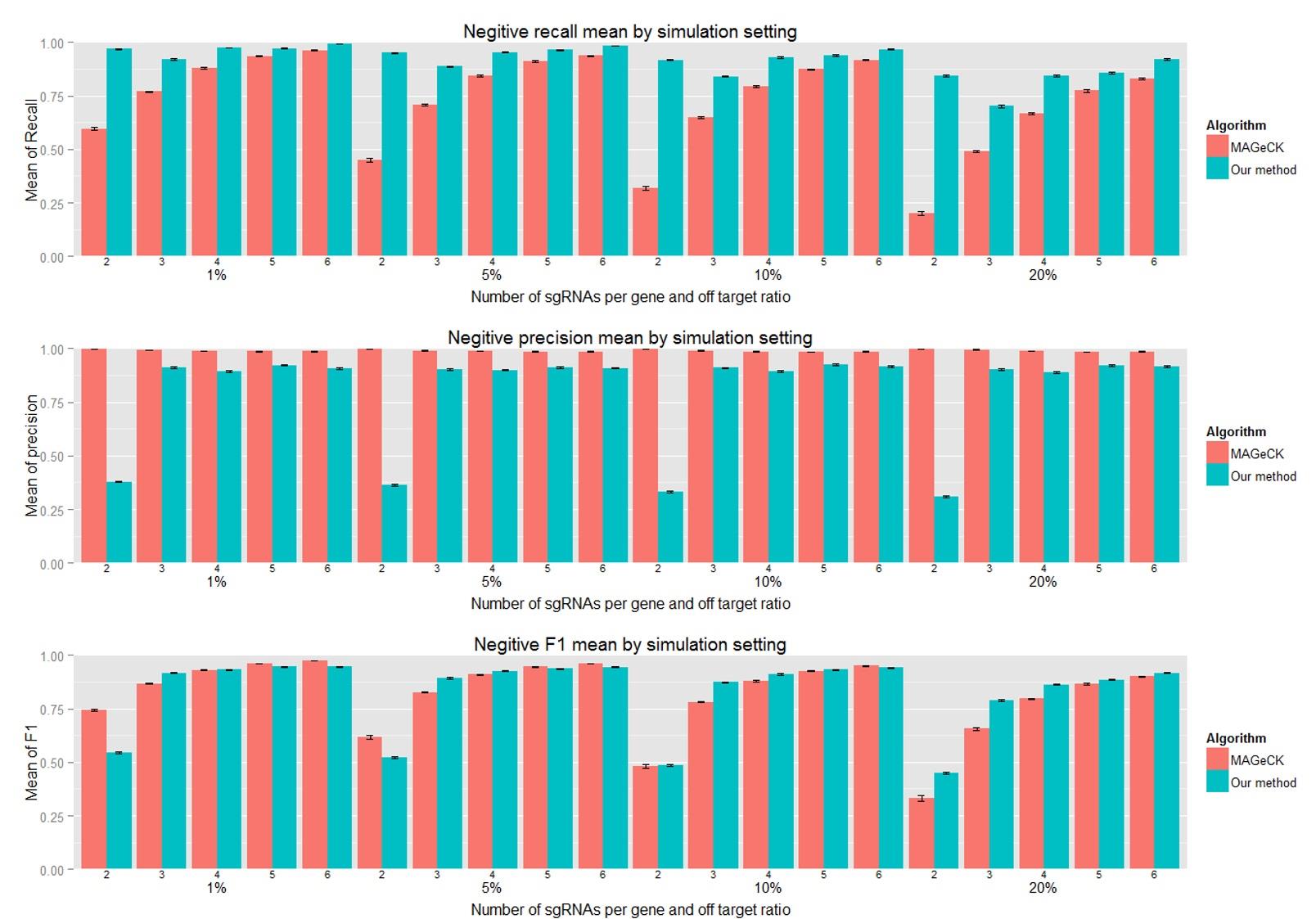


Figure S5 Simulation evaluation of negative selection performance based on recall, precision and $F_{1}$ for different combinations of sgRNA number per gene (2~6) and off target ratio. Each bar represents the average of 50 simulated datasets and standard error is indicated on the bar.
